# Supplementary material for: Single-cell transcriptional profiling of clear cell renal cell carcinoma reveals a tumor-associated endothelial tip cell phenotype
Source: Commun Biol. 2024 Jun 28;7:780. doi: 10.1038/s42003-024-06478-x (PMC11213875; doi:10.1038/s42003-024-06478-x)
Supplement: Supplementary file 1 — Supplementary Information [file 42003_2024_6478_MOESM1_ESM.pdf]

Supplementary Information

Single-cell transcriptional profiling of clear cell renal cell carcinoma reveals a tumor associated endothelial tip cell phenotype

Justina Zvirblyte[1], Juozas Nainys[1,§], Simonas Juzenas[1], Karolis Goda[1], Raimonda Kubiliute[2], Darius Dasevicius[3], Marius Kincius[4], Albertas Ulys[4], Sonata Jarmalaite[2,4,*] and Linas Mazutis[1,*]

[1]Institute of Biotechnology, Life Sciences Center, Vilnius University, Vilnius, 10257, Lithuania

[2] Institute of Biosciences, Life Sciences Center, Vilnius University, Vilnius, 10257, Lithuania

[3] National Center of Pathology, Affiliate of Vilnius University Hospital Santaros Klinikos Vilnius, 08406, Lithuania

[4] National Cancer Institute, Vilnius, 08660, Lithuania

[§] Current address: Atrandi Biosciences, Vilnius, 10257, Lithuania,

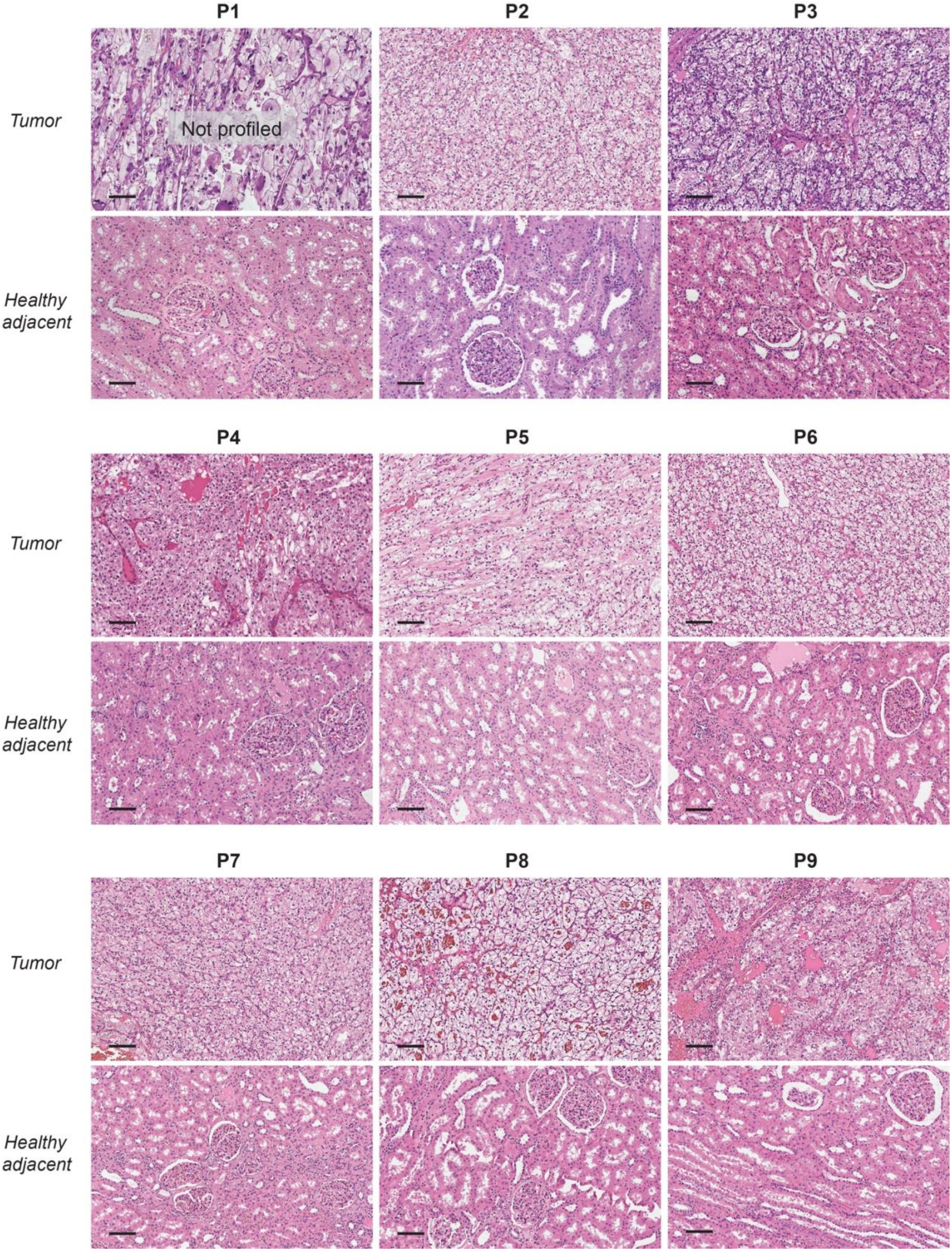

Supplementary figure S1. H&E staining histology slides for tissues analyzed in the study. Slides presented in pairs – tumor and healthy adjacent tissue staining for patient P1, P2 etc. All tumor samples present with WHO/ISUP differentiation grade II, except for P1 and P4, which are of grade IV. Note: tumor sample from patient P1 was not profiled due to low cell viability after dissociation. Scale bar – 100 μm.

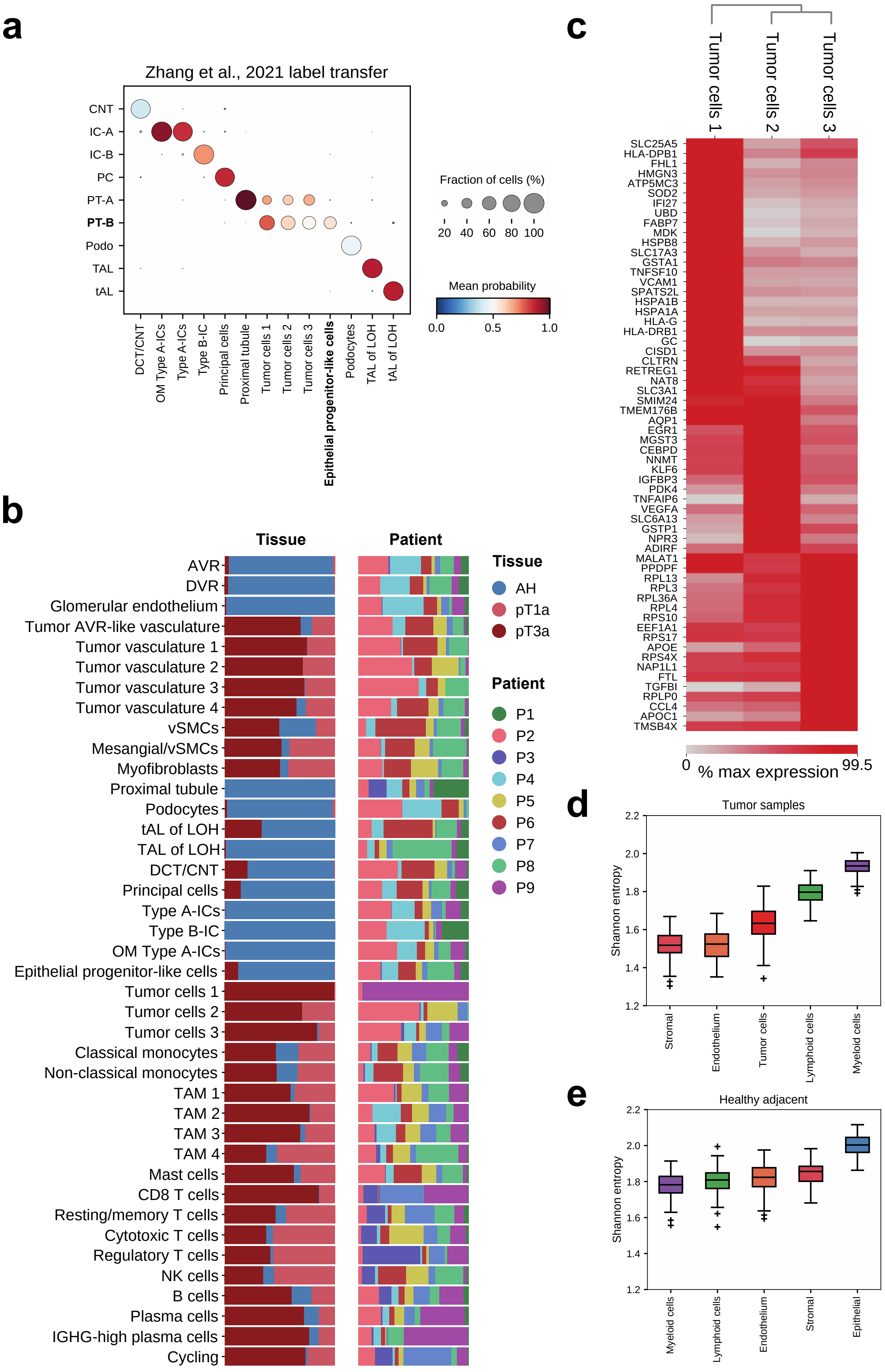

a
Zhang et al., 2021 label transfer
CNT
IC-A
IC-B
PC
PT-A
PT-B
Podo
TAL
tAL
DCT/CNT
OM Type A-ICs
Type A-ICs
Type B-IC
Principal cells
Proximal tubule
Tumor cells 1
Tumor cells 2
Tumor cells 3
Epithelial progenitor-like cells
Podocytes
TAL of LOH
tAL of LOH
Fraction of cells (%)
20 40 60 80 100
Mean probability
0.0 0.5 1.0
b
Tissue
Patient
AVR
DVR
Glomerular endothelium
Tumor AVR-like vasculature
Tumor vasculature 1
Tumor vasculature 2
Tumor vasculature 3
Tumor vasculature 4
vSMCs
Mesangial/vSMCs
Myofibroblasts
Proximal tubule
Podocytes
tAL of LOH
TAL of LOH
DCT/CNT
Principal cells
Type A-ICs
Type B-IC
OM Type A-ICs
Epithelial progenitor-like cells
Tumor cells 1
Tumor cells 2
Tumor cells 3
Classical monocytes
Non-classical monocytes
TAM 1
TAM 2
TAM 3
TAM 4
Mast cells
CD8 T cells
Resting/memory T cells
Cytotoxic T cells
Regulatory T cells
NK cells
B cells
Plasma cells
IGHG-high plasma cells
Cycling
Tissue
AH
pT1a
pT3a
Patient
P1
P2
P3
P4
P5
P6
P7
P8
P9
c
Tumor cells 1
Tumor cells 2
Tumor cells 3
SLC25A5
HLA-DPB1
FHL1
HMGN3
ATP5MC3
SOD2
IFI27
UBD
FABP7
MDK
HSPB8
SLC17A3
GSTA1
TNFSF10
VCAM1
SPATS2L
HSPA1B
HSPA1A
HLA-G
HLA-DRB1
GC
CISD1
CLTRN
RETREG1
NAT8
SLC3A1
SMIM24
TMEM176B
AQP1
EGR1
MGST3
CEBPD
NNMT
KLF6
IGFBP3
PDK4
TNFAIP6
VEGFA
SLC6A13
GSTP1
NPR3
ADIRF
MALAT1
PPDPF
RPL13
RPL3
RPL36A
RPL4
RPS10
EEF1A1
RPS17
APOE
RPS4X
NAP1L1
FTL
TGFBI
RPLP0
CCL4
APOC1
TMSB4X
0 % max expression 99.5
d
Tumor samples
Shannon entropy
2.2
2.0
1.8
1.6
1.4
1.2
Stromal
Endothelium
Tumor cells
Lymphoid cells
Myeloid cells
e
Healthy adjacent
Shannon entropy
2.2
2.0
1.8
1.6
1.4
1.2
Myeloid cells
Lymphoid cells
Endothelium
Stromal
Epithelial

Supplementary figure S2. Cell composition and inter-patient variability in ccRCC. **a**) CellTypist model label transfer from Zhang et al. 2021 dataset. X-axis labels are cell populations from this study and y-axis labels are from Zhang et al., 2021.The annotation of cell types in both studies is in agreement and epithelial progenitor-like cell population is most similar to cell-of-origin ‘PT-B’ cell population from Zhang et al., 2021. **b**) Cell composition by disease stage and patient ID. Specialized epithelial and endothelial cells originate mostly from the healthy-adjacent tissues, while immune, tumor, endothelial and stromal cells are enriched in the tumor samples. Different types of cells are adequately represented by multiple samples, except for tumor cells 1 population, which appears specific to patient P9. **c)** Differential gene expression between tumor cell subpopulations. Only genes with Benjamini-Hochberg adjusted p-value <0.05 are shown. **d, e**) Tumor and healthy adjacent sample heterogeneity for broad cell group as measured by Shannon entropy. Lower entropy values indicate higher sample heterogeneity. AH – adjacent healthy.

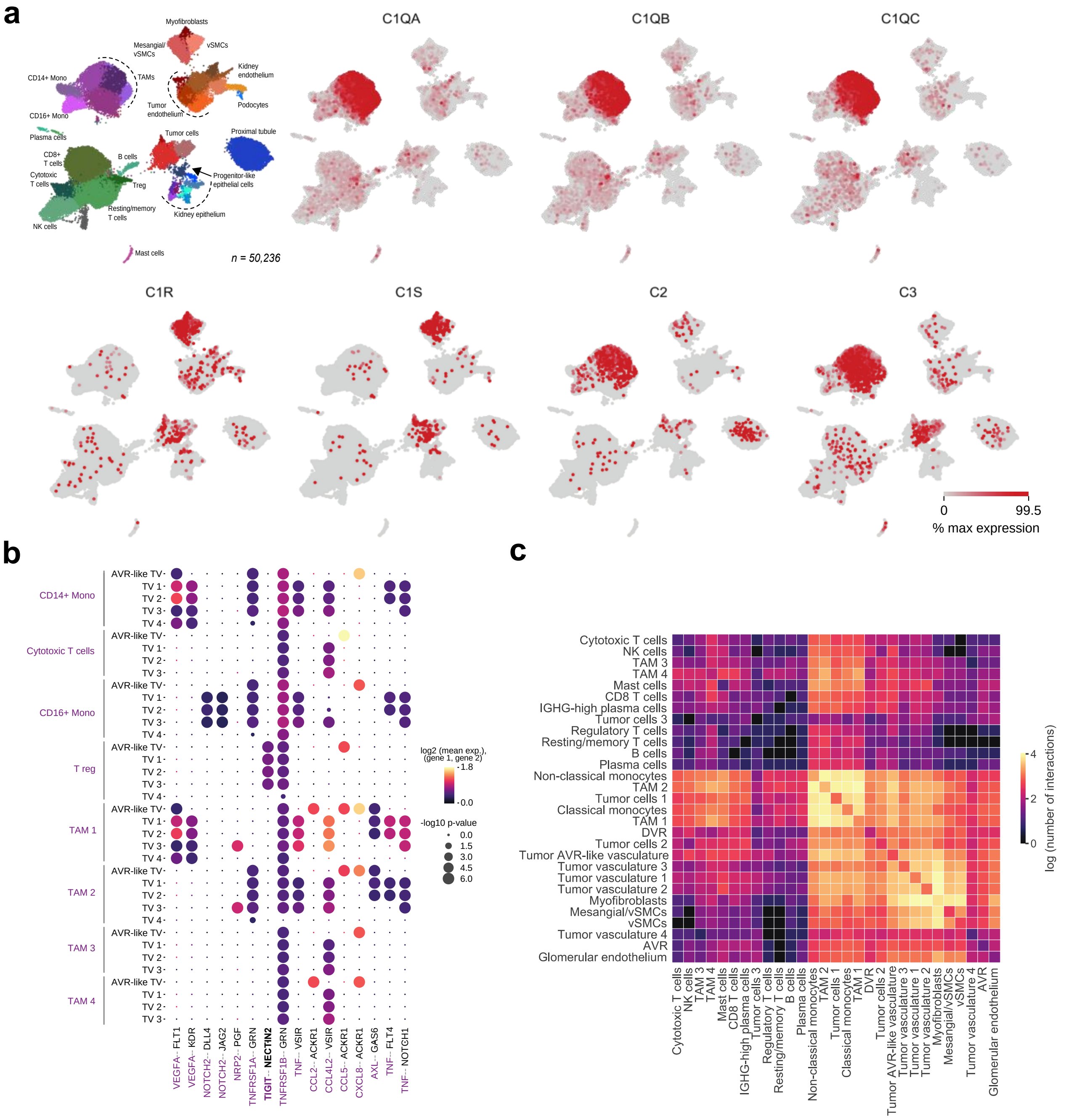

Supplementary figure S3. Expression of complement system genes and cell-cell communication analysis. **a)** Expression of complement system molecules C1QA, C1QB and C1QC is specific to tumor associated macrophages, while C1R, C1S are expressed by tumor and stromal cells. **b)** Cell-cell communication analysis between immune cells and tumor vasculature reveal immunosuppressive TIGIT-NECTIN2 interaction between tumor vasculature and regulatory T cells. **c)** Count matrix of all major cell-cell interactions observed within the TME of ccRCC

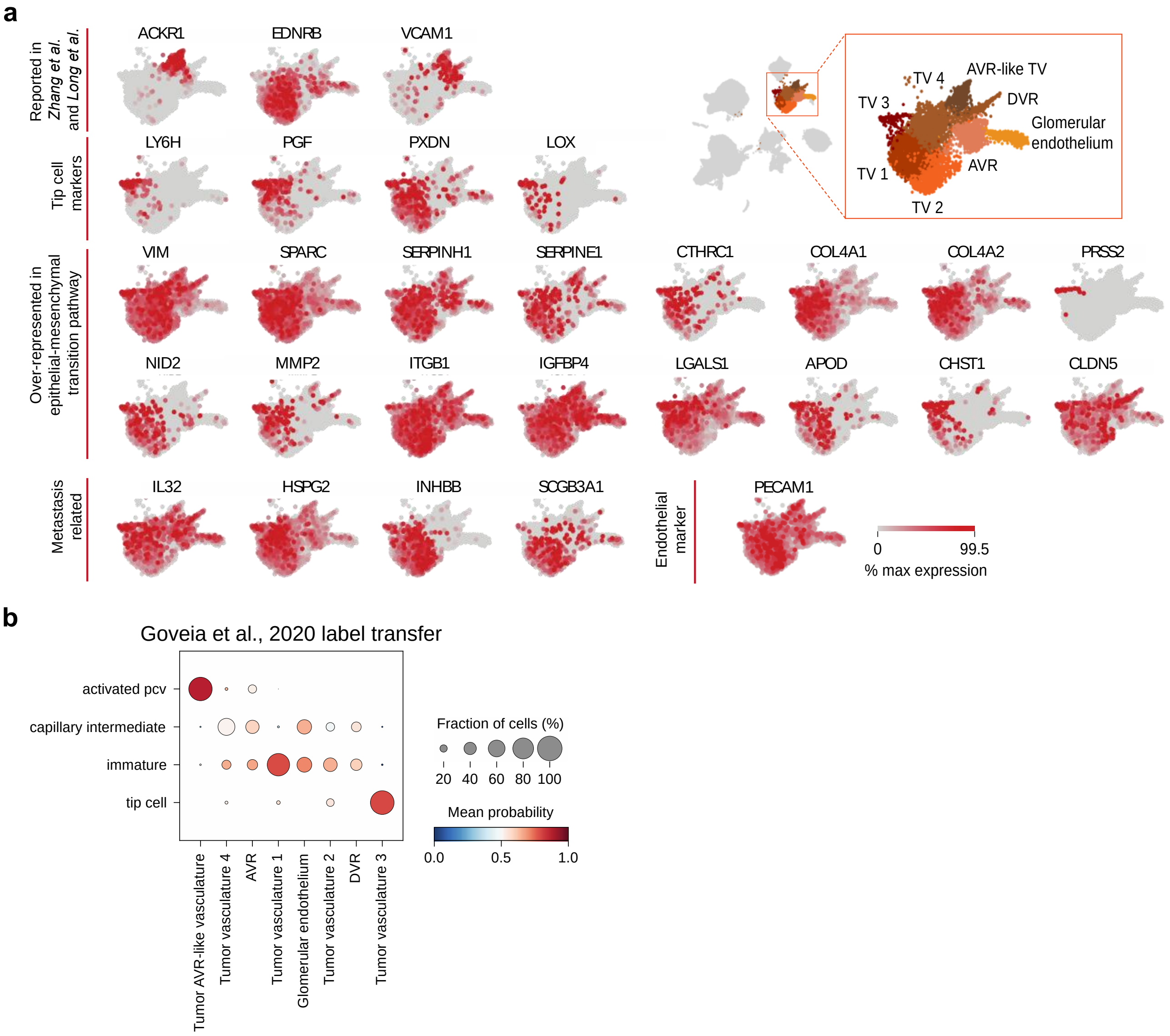

Supplementary figure S4. Tumor endothelial cell characterization. **a)** Expression of metastasis associated genes, tip-cell markers and genes overlapping with epithelial-mesenchymal transition pathway in tumor vasculature. The tip-cell markers are mostly enriched in tumor vasculature 3 population, while other genes are expressed in multiple tumor vasculature populations in heterogeneous manner. Previously reported markers *ACKR1*, *EDNRB* and *VCAM1* are not expressed in tumor vasculature 3. **b)** CellTypist model label transfer from Goveia et al. 2020 dataset. X-axis labels are cell populations from this study and y-axis labels are from Goveia et al., 2020. This analysis suggests that tumor vasculature populations in our study represent immature vessels described by Goveia et al. 2020, and Tumor vasculature 3 population represents the tip cell phenotype. AVR – ascending vasa recta, DVR – descending vasa recta, pcv – post-capillary vein, TV – tumor vasculature.

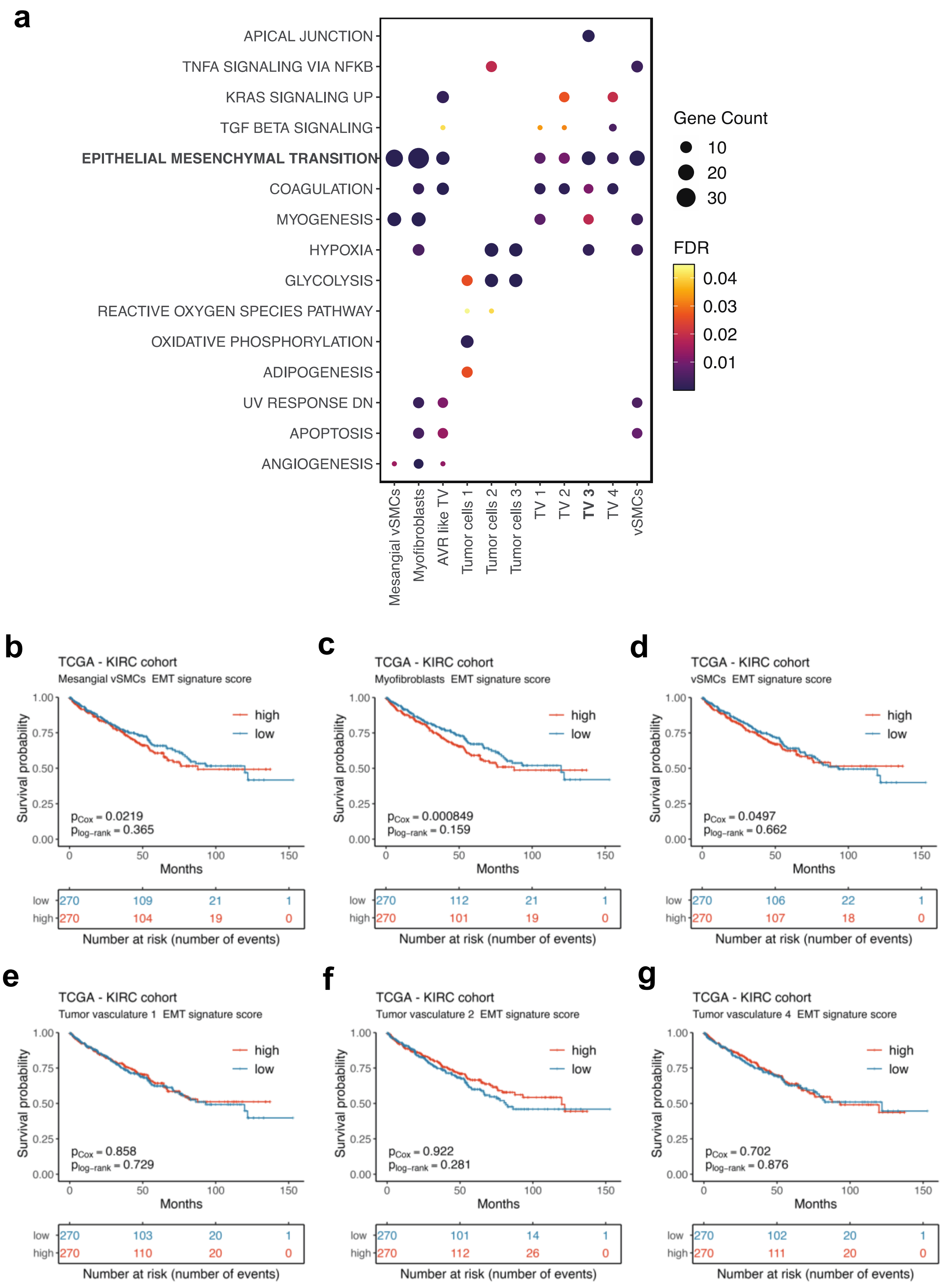

Supplementary figure S5. MSigDB Hallmark pathway overrepresentation analysis. **a)** Cell type signature over-representation analysis reveals enrichment of epithelial-mesenchymal transition (EMT) in tumor vasculature and stromal cell populations. **b-g)** None of the stromal and tumor vasculature signature genes overlapping with EMT correlate with overall survival in the TCGA KIRC cohort (except for tumor AVR-like vasculature and tip-like tumor vasculature 3, as shown in Figure 4b-c).

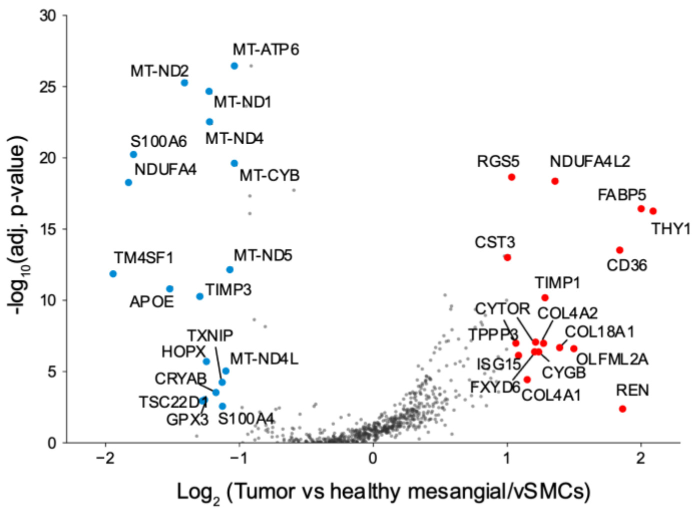

Supplementary figure S6. Volcano plot of differentially expressed genes between tumor originating and healthy-adjacent originating cells in mesangial/vSMC population. Genes with fold-change of 2 and adjusted p-value <0.05 are highlighted and considered significant. The asymmetry of the central position of the volcano plot is due to difference in cell size with tumor cells having higher fraction of non-zero genes. This effect was not corrected to maintain consistency in differential gene expression analysis performed.

Table 1. Cluster annotation by top 25 DEGs.

Cluster No in Supp. Table S2	Annotation	Top 25 genes and references
0	Tumor vasculature 1	PLVAP[1-3], SPRY1, SPARC, PLPP1, COL4A1[4], VWA1, SPARCL1[5,6], VWF[2,4,7], HSPG2[2], PLPP3[4], GNG11, COL4A2[4], RGCC, RBP7, IGFBP7[4], TIMP3[8], RAMP2[8], IFI27, EGFL7[9], FLT1[1,2], RAMP3, ESAM, INSR[4], GSN, A2M
1	Tumor vasculature 2	IGFBP3[10], ENPP2[7], RBP7, ESM1, SPARCL1[5,6], FLT1[1,2], A2M, PLVAP[1-3], SPRY1, STC1, ANGPT2, INSR[4], PRSS23, PLPP3[4], VWF[2,4,7], PLPP1, GNG11, PECAM1[1,2,8], SPARC, EFNB2[11], GJA1, TIMP3[8], IGFBP7[4], IFI27, EPAS1[12]
2	Proximal tubule	MIOX[8,13], ALDOB[8], MT1G[8,13], GATM[8,13], GSTA2[8], GSTA1[8], ASS1[8,14], FABP1[8], NAT8[8], GPX3[8,14], PDZK1IP1[2,8,13], PCK1[8], MT1H[8], RBP4[8], DCXR[8], HPD[8], SUCLG1[8], ECHS1[8], FBP1[8], UGT2B7[8], BBOX1[8], GLYAT[8], RIDA[8], PEPD[8], CYB5A[8]
3	Plasma cells	IGHA1[15,16], IGLC3[16], IGHM[16], IGLC2[15,16], JCHAIN[16], IGKC[16], MZB1[15,16], SSR4, DERL3, SEC11C, FKBP11, CD79A[15,16], HERPUD1, PRDX4, HSP90B1, XBP1, FKBP2, ITM2C, DNAAF1, CD27, SELENOS, ATF4, LMAN2, SDF2L1, SSR3
4	TAM 1	CCL3L1, CCL3[2,17,18], IL1B[2,19,20], CXCL8[17,20,21], IER3, CCL4L2, TNF[19,20], CXCL3[17], CXCL2[17], MS4A7, BCL2A1, C1QC[22], PLAUR, C1QA[20,22,23], HLA-DRA[20], GPR183, EIF4E, C1QB[20,22], CD83, NFKBIA, HLA-DPA1[20], HLA-DPB1[20], CCL20[2], CD74[20,23], HLA-DQB1[20]
5	Principal cells	FXYD4[11,13], AQP2[8,11,24], AQP3[8,11,24], GDF15, WFDC2, EPCAM, HSD11B2[11,13], KRT19[13], TACSTD2, CLU[25], CDH16, SLPI[13], AIF1L, ELF3, DEFB1[14], CLDN4[8,26], ATP1B1, RASD1, FOLR1, S100A2, COBLL1, GSTM3, RALBP1, SPINT2, CD24
6	vSMCs	TAGLN[11,13,27], ACTA2[11,13,27], MYL9, ADIRF, TPM2, RGS5, CALD1, C11orf96, PLN, REN, MYH11[11,13], MGP, RERGL[11], BGN, PPP1R14A, PLAC9, CSRP2, SOD3, DSTN, RGS16, NOTCH3[27], IGFBP7, MFGE8, MYLK, CCL2
7	Glomerular endothelium	IGFBP5[4,8,28], EMCN[8,11,13], CRHBP[2,8], PLAT[8,11], SOST[2,8], SLC9A3R2[8,28], EHD3[8,11], MGP[8], PLPP3[8], RNASE1[8], IFI27[8], TGFBR2[8], FCN3[8], PLPP1[8], ID1[8], RAMP2[8], GNG11[8], IFITM3[8], ADGRF5[8], PTPRB[8], SLC14A1[8], EPAS1[8], MEIS2[8], TIMP3[8], APP[8]
8	Classical monocytes (CD14+)	S100A9[15,20,29], S100A8[15,20,29], LYZ[29], G0S2, FCN1[15], IL1B, EREG, THBS1, CXCL8, S100A12[15,29], VCAN[15,29], PLAUR, CXCL2, BCL2A1, CTSS, AREG, C5AR1, NAMPT, PPIF, LST1, NLRP3, IER3, SOD2, CEBPB, ATP2B1-AS1
9	Mast cells	TPSB2[8], TPSAB1[8,20], CPA3[8], AREG[8], CD69[8], CTSG[8], MS4A2[8], LMNA[8], LTC4S[8], HPGDS[8], RGS13[8], RHEX, RGS2, KIT[20], HPGD, VWA5A, SRGN, FOSB, CPM, GATA2, NFKBIA, GPR65, ANXA1, LMO4, PPP1R15A
10	Distal convoluted tubule/connecting tubule	DEFB1[11,14], CALB1[11,13,30], HSD11B2, KLK1[11], KNG1, ATP1B1, TMEM52B[13], CA12, MAL, WFDC2[8], SLC12A3[11,13,30], RHCG[8], GDF15, NFE2L2[8], EPCAM, CDH16, HMGCS2, FXYD2[11,14], SERPINA5, CA2, KCNJ1[1,31], KRT19, MTRNR2L8, EMX1, CLDN4[8]

11	Mesangial/vSMCs	RGS5, BGN[25,32,33], REN[34], PLAC9, ADIRF, IGFBP7, CALD1[33], MGP[13], MYL9[33], NDUFA4L2, HIGD1B, TPM2, ID3, FABP4, NOTCH3, CD36[35], PDGFRB[11,13,27], C11orf96, CSRP2, FRZB, TAGLN[11], TPPP3, THY1[30], COL1A2, LHFPL6
12	Mast cells	TPSB2[8], TPSAB1[8,20], CPA3[8], CTSG[8], CD69[8], AREG[8], HPGDS[8], RHEX, MS4A2[8], HPGD, VWA5A, CCL2, RGS13[8], RGS2, LTC4S, SRGN, ANXA1, LMNA, GPR65, KIT[20], ALOX5AP, PPP1R15A, LAPTM4A, FCER1A, AC020571.1
13	Cycling	HIST1H4C, STMN1[8], HMGB2[36], PCLAF, UBE2C[8,36], HMGN2, TUBB, CKS1B, MKI67[8,23,36], TUBA1B, H2AFV, H2AFZ, CENPF[36], PTTG1, TYMS[8,36], RRM2[36], HMGB1, PCNA[36], DUT, CLSPN[36], NUSAP1[36], GZMK, GZMA, CXCL13, DEK
14	IGHG-high plasma cells	IGKC[16], IGHG3, IGHG1, IGLC2[15,16], IGHG4, IGLC3[16], JCHAIN[16], MZB1[15,16], IGHGP, IGHG2, SSR4, SEC11C, XBP1, CD79A[15,16], FKBP11, DERL3, HSP90B1, ITM2C, PRDX4, FAM30A, ERLEC1, SPCS1, IGHM, DNAAF1, KLF13
15	Non-classical monocytes (CD16+)	LST1[37], SMIM25, FCN1[15], AIF1[37], G0S2, LYPD2[38], COTL1, SAT1, FCER1G, CTSS[37], BCL2A1, IL1B, LILRB2[15], PLAUR, FCGR3A[20,37,39], C5AR1, TYROBP, MS4A7, TIMP1, NAP1L1, POU2F2, IFI30, STXBP2, CALHM6, NEAT1
16	Descending vasa recta	TIMP3[8], CLDN5[2,13], AQP1[2,11,40], SLC9A3R2[28], TM4SF1, IFI27, RAMP2[8], RBP7, SLC14A1[11,13,40], DEPP1, ID1[2], CRIP2, SERPINE2[2], PPP1R14A, SRP14, PALMD[13], FAM107A, PLPP1, ICAM2, RNASE1, SSUH2, KCTD12, S100A6, ABI3BP, IFITM3[8]
17	Proximal tubule	MT1G[8,13], ALDOB[8], MT1H[8], GPX3[8,14], FABP1[8], PDZK1IP1[2,8,13], MIOX[8,13], HPD[8], MT1X[8], CXCL14[8], ASS1[8,14], GATM[8], NAT8[8], MT1F[8], GSTA1[8], SUCLG1[8], SMIM24[8], FXYD2[8,13], RBP5[8], RIDA[8], MT1E[8], SPP1[8], UGT2B7[8], LGALS2[8], ALB
18	Tumor vasculature 3	LY6H, CLDN5[2,13], PGF, COL4A1[4], APOD, SPARC[41], COL4A2[4], HSPG2[2], CHST1, TNFRSF4, PXDN, VWF[2,4,7], LAMA4, GNG11, CCDC85B, PECAM1[1,2,8], SPARCL1[2], PRSS2, FSCN1[4], SPRY1, CRIP2, LXN, ICAM2, TCF4, CD93
19	Tumor cells 1	FABP7[42], GC, CD24[43,44], ANXA4, SLC17A3[2,23], NDRG1[45], UBD, PLIN2[46], CRYAB[23], CLU[23], SPATS2L, FHL1, MDK, HLA-G, MGST2, GSTA1, HMGN3, CISD1, ATP1B1, NNMT[2], SLC13A1, CXCL14, SOD2[23], HSPB8, VCAM1[23]
20	Outer medulla type A intercalated cells	SPINK1[8], TMEM213[8,13], ATP6V1G3[11,36,47], SLC4A1[1,11,13], DEFB1, ATP6V0D2[11,13,36], RTN4[8], CKB[8], RHCG[8], EPCAM, SMIM6, ATP6AP2[8,13], MAL[8], FAM24B[8], CA12[8], LGALS3, HSD11B2, C12orf75[13], ADGRF5[11,13,47], ATP6V0B[8], BSG, DHRS7, ATP6V0A4, CLCNKB[1,11], ATP1B1<br>Also positive for marker SLC26A7[48]
21	Myofibroblasts	COL1A1[49], COL1A2[49], TIMP1[49], MGP[8], TAGLN[8], COL3A1[49], ACTA2[1,8,49], BGN[8,10], FN1, TPM2[8], DCN[10], CALD1[8,10], MYL9[8], IGFBP7[8], POSTN, RGS5[8], LUM[49], COL6A2[8], SPARC[8,10], PLAC9[8], COL4A2, AEBP1, COL6A1[10], PPP1R14A, COL4A1

22	Tumor cells 2	NNMT[2], NDUFA4L2[2,23,50], CD24[43,44], VEGFA[23], PDK4, CRYAB[23], ANGPTL4[2,51], PLIN2[46], CLU[23], TMEM176B, NDRG1[45], TMEM176A, RARRES2, HILPDA, BNIP3[52], ANXA4, RNASET2, CYB5A, CCDC146, KRT8, CCND1[52], ENO1, CXCL14, KRT18, EGR1
23	Thick ascending limb of LOH	DEFB1[8,11], UMOD[11,14,30], PCP4[8], FXYD2[8,14,31], KNG1[8,13], CKB[8], GSTM3[8], MPC1[8], LDHB[8], PEBP1[8], MRPS6[8], PPP1R1A[8], CYSTM1, BEX3, SLC25A5[8], SLC12A1[8,11,14], CBR1, ATP5F1A[8], SLC25A4[8], CD24[8], TCIM, ATP5MC3[8], UQCRFS1[8], PRDX2[8], MAL
24	Tumor AVR-like vasculature	ACKR1[1,2,8], VWF[2,4,7], RNASE1[8], CLU[2], IFI27[8], TFF3, CD59, HYAL2[8], RAMP3[8], FKBP1A, FN1, TM4SF1[8], IFITM3[8], PTGDS, PECAM1[1,2,8], ADIRF, DNASE1L3[2,13], S100A6, IGFBP4[8], SLCO2A1[13], ECSCR, FAM167B, RAMP2[8], IL33, TGM2
25	Regulatory T cells	LTB, TNFRSF18[23,53], IL32, BATF[53], TIGIT[53,54], CARD16, CORO1B, TNFRSF4[53,54], TRAC[23], TRBC1, S100A4, LINC01943, FOXP3[23,53,54], CD3D[41,53], LAIR2, AC133644.2, LINC02195, CD27, CTLA4[53,54], PMAIP1, TBC1D4, DUSP4, CYTIP, SPOCK2, ICA1
26	Thick ascending limb of LOH	UMOD[11,14,30], DEFB1[8,11], KNG1[8,13], SLC12A1[8,11,14], ATP1A1, ATP1B1, S100A2, CLDN10, SFRP1, KCNJ1[8,11], CA12, MAL, CKB[8], TMEM52B, CDH16, UCHL1, GSTM3, PCP4[8], SERPINA5, CLCNKB, TSPAN8, MPC1[8], SPP1, MTRNR2L8, EGF
27	Tumor cells 3	FABP7[42], NDUFA4L2[2,23,50], CD24[43,44], NNMT[2], BNIP3[52], HILPDA, CRYAB[23], PLIN2[46], LDHA[52], RARRES2, ANGPTL4[2,51], ANXA4, GAPDH, ENO1, TPM1, NDRG1[45], CLU[23], TPI1, VDAC1, SERPINA1, TMEM176A, NUPR1, KRT18, VEGFA[23], EGLN3[55]
28	Tumor vasculature 4	PLVAP[1-3], GNG11, SPARC, RBP7, SPRY1, HSPG2[2], VWF[2,4,7], IFI27, RAMP2[8], PLPP1, COL4A1[8], SPARCL1[5,6], IGFBP7[4], TIMP3[8], MTRNR2L8, EGFL7[9], INSR[4], GSN, RAMP3, ESM1, VWA1, IFITM3[8], ECSCR, TCF4, SLC9A3R2
29	Podocytes	PTGDS[8], DCN[8], NPHS2[8,11,13], EMCN, IGFBP5, TNNT2[8], IGFBP2[8], HTRA1[8], PCOLCE2[8], CTGF, PLAT, PODXL[8,13,56], TPPP3[8], MYL9[8], CRHBP, APOD[8], MME[8], HPGD, CLIC5[8,13,56], TCF21[8,56], CDKN1C[11,47], SLC9A3R2, ID1, BST2[8], AIF1[8]
30	TAM 2	APOC1[39], APOE[20,23,39], C1QB[20,22], C1QA[20,22,23], C1QC[22], CTSD[39], HLA-DRA[20], TYROBP, TREM2[54], HLA-DPA1[20], HLA-DQA1[20], HLA-DRB1[20], FCER1G, PSAP, HLA-DPB1[20], LYZ, HLA-DQB1[20], CTSB, GPNMB[23,39], NPC2, CST3, CD68[1,2,54], CD74[20,23], HLA-DRB5[20], CFD
31	CD8 T cells	DUSP4[41], GZMK[41,53], CST7, LYST, CCL5[53], TRBC2, CD8B[53], CD3D[41,53], RGS1, CD27[41], CREM[41], RGS2[41], NKG7[53], TRAC[53], TNFRSF9[41,53], CD8A[41,53], TRBC1, HSP90AA1[41], GZMA[23], SRSF7, CD2, PMAIP1, CMC1, RUNX3[57], SYTL3
32	Type A intercalated cells	ATP6V1G3[11,36,47], DEFB1, TMEM213[8,13], C12orf75[8], SPINK1[8], CKB[8], FXYD2[8], MAL[8], ATP6V0D2[11,13,36], FAM24B[8], SMIM6, NUPR2, LGALS3[8], CYSTM1[8], CA12[8], CLCNKB[1,11], RTN4[8], SLC25A5[8], SLC25A39[8], BSG[8],

		HOXB-AS3, ERP27[13], COX7A1, SLC4A1[1,11,13], ADGRF5[11,13,47]
33	B cells	CD79A[15,16,23], CD37[16], IGHM, IGKC, MS4A1[15,16], LTB[16], BANK1, IGHD, LINC01781, LINC00926, CD83, RALGPS2, FAM30A, CD52, BIRC3, EEF1B2, RPS5, POU2F2, SPIB, RPL32, RPL18A, LINC01857, RPS8, CD79B[15,16], CD55
34	Thin ascending limb of LOH	WFDC2, S100A2[58], SLPI, SOD3, PAPPA2[59], MMP7, DEFB1, ITM2C, TACSTD2[13], MAL, SLC12A1[13], PCSK1N, IGFBP6, CLDN10[13,58], S100A6[58], UMOD[13,58], CLU[14], TSPAN8[13], CA12, MUC1, CD24, CLDN3, ATP1A1, KRT7, EPCAM
35	Ascending vasa recta	DNASE1L3[2,13], IGFBP5[28], RNASE1[8], RAMP3[8], EMCN[8,13], CAVIN2[8], IFI27[8], ID1, TMEM88, RAMP2[8], SLC9A3R2[28], CA4, GNG11[8], IFITM3[8], FAM167B, ENG[8], TIMP3[8], TM4SF1[8], PLPP3, HYAL2[8], PCAT19, IGFBP4[8], PLAT[8], MEIS2[8], GIMAP7
36	Type B intercalated cells	KRT7[8], WFDC2[8], ATP6V1G3[11,36,47], TMEM213[8,13], S100A2[8], CD9[8], CA12[8], MTRNR2L12, DEFB1, TSPAN8[8], MAL[8], RARRES2[8], CDA[8], ATP6V0B[8], ATP6V0D2[8,11,13], ATP6AP2[8], LGALS3[8], ATP6V0A4[8], ATP6V1B1[8,11], MTRNR2L10, SMIM24[8], EPCAM, CKB, MTRNR2L1, MTRNR2L8<br>Also positive for markers: SLC26A4[11,13,60], HMX2[11], SPINK8[11]
37	TAM 3	HLA-DPB1[20], HLA-DPA1[20], APOC1[39], CST3, HLA-DRA[20], TYROBP, AIF1, HLA-DQA1[20], C1QA[20,22,23], HLA-DQB1[20], C1QB[20,22], CD74[20,23], MS4A6A, C1orf162, FTL, LYZ, RGS10, NPC2, CD68[1,2,54], LST1, C1QC[22], SAT1, IER3, HLA-DMA[20], HLA-DRB1[20]
38	Epithelial progenitor-like cells	CRYAB[8], SLPI[8], MMP7[8], CLU[8,61], CLDN4[8], WFDC2[8], TACSTD2[8], CD24[8,58,61], CTGF, CLDN3[8], NUPR1, MT1E, ELF3[8], EGOT, TPM1[8,58], KRT19, MAL[8], PIGR, S100A13[8], SPINT2[8], SOX4[61], RASD1, ATF3, MT1X, BCAM[8]
39	Cytotoxic T cells	XCL1[53], XCL2[53], KLRB1[62], TRDC, KLRC1, KRT86, CD7, KLRD1[62], HOPX[53], GNLY[53,62], CCL4[53], KRT81, CCL5[53], ZNF683[53], LINC01871, CTSW[62], HCST, IL2RB, AREG, ZFP36L2, BTG1, REL, FAM177A1, MATK, CMC1
40	TAM 4	C1QB[20,22], C1QA[20,22,23], C1QC[22], SELENOP[20], FOLR2[20,23], RNASE1, HLA-DPA1[20], CD14, HLA-DRA[20], MS4A6A, MS4A7, HLA-DRB1[20], AIF1, HMOX1, MS4A4A[20], TYROBP, HLA-DPB1[20], NPC2, CD74[20,23], HLA-DQA1[20], CST3, HLA-DQB1[20], FCGRT, SLC40A1[21,39], FCER1G, PLTP[20], BLVRB
41	NK cells	GNLY[63], GZMB[63], FGFBP2, NKG7[63], KLRD1[63], KLRB1[53], PRF1[63], GZMH[63], SPON2[63], PLAC8, CLIC3, KLRF1[63], CD247[63], AREG, GZMM, HOPX, CST7[63], TRDC, CMC1[63], ARL4C, CTSW, CCL4[63], PTGDS[63], GZMA[63], EFHD2
42	Resting/memory T cells	IL7R[23,53], CD52[53], LTB[64], KLRB1[53], ZFP36L2[41], RPS29, CD48, CXCR4[41], LYAR, BTG1, ANXA1, STK4, RPS27, EML4, PPP2R5C, RPS3, ACAP1, RPS15A, SPOCK2, ISG20, FYN, CD3D[41,53], PDE4B, CD2, RPLP2

Supplementary References:

1 Young, M. D. *et al.* Single-cell transcriptomes from human kidneys reveal the cellular identity of renal tumors. *Science* **361**, 594-599, doi:10.1126/science.aat1699 (2018).

2 Zhang, Y. *et al.* Single-cell analyses of renal cell cancers reveal insights into tumor microenvironment, cell of origin, and therapy response. *Proc Natl Acad Sci U S A* **118**, doi:10.1073/pnas.2103240118 (2021).

3 Strickland, L. A. *et al.* Plasmalemmal vesicle-associated protein (PLVAP) is expressed by tumour endothelium and is upregulated by vascular endothelial growth factor-A (VEGF). *J Pathol* **206**, 466-475, doi:10.1002/path.1805 (2005).

4 Dumas, S. J. *et al.* Phenotypic diversity and metabolic specialization of renal endothelial cells. *Nat Rev Nephrol* **17**, 441-464, doi:10.1038/s41581-021-00411-9 (2021).

5 Gagliardi, F., Narayanan, A. & Mortini, P. SPARCL1 a novel player in cancer biology. *Crit Rev Oncol Hematol* **109**, 63-68, doi:10.1016/j.critrevonc.2016.11.013 (2017).

6 Naschberger, E. *et al.* Matricellular protein SPARCL1 regulates tumor microenvironment-dependent endothelial cell heterogeneity in colorectal carcinoma. *J Clin Invest* **126**, 4187-4204, doi:10.1172/JCI78260 (2016).

7 Su, S. C. *et al.* Autotaxin-lysophosphatidic acid signaling axis mediates tumorigenesis and development of acquired resistance to sunitinib in renal cell carcinoma. *Clin Cancer Res* **19**, 6461-6472, doi:10.1158/1078-0432.CCR-13-1284 (2013).

8 Stewart, B. J. *et al.* Spatiotemporal immune zonation of the human kidney. *Science* **365**, 1461-1466, doi:10.1126/science.aat5031 (2019).

9 Hirsch, L., Flippot, R., Escudier, B. & Albiges, L. Immunomodulatory Roles of VEGF Pathway Inhibitors in Renal Cell Carcinoma. *Drugs* **80**, 1169-1181, doi:10.1007/s40265-020-01327-7 (2020).

10 Wu, H. *et al.* Single-Cell Transcriptomics of a Human Kidney Allograft Biopsy Specimen Defines a Diverse Inflammatory Response. *J Am Soc Nephrol* **29**, 2069-2080, doi:10.1681/ASN.2018020125 (2018).

11 Balzer, M. S., Rohacs, T. & Susztak, K. How Many Cell Types Are in the Kidney and What Do They Do? *Annu Rev Physiol* **84**, 507-531, doi:10.1146/annurev-physiol-052521-121841 (2022).

12 Cancer Genome Atlas Research, N. Comprehensive molecular characterization of clear cell renal cell carcinoma. *Nature* **499**, 43-49, doi:10.1038/nature12222 (2013).

13 Lake, B. B. *et al.* A single-nucleus RNA-sequencing pipeline to decipher the molecular anatomy and pathophysiology of human kidneys. *Nat Commun* **10**, 2832, doi:10.1038/s41467-019-10861-2 (2019).

14 Lee, J. W., Chou, C. L. & Knepper, M. A. Deep Sequencing in Microdissected Renal Tubules Identifies Nephron Segment-Specific Transcriptomes. *J Am Soc Nephrol* **26**, 2669-2677, doi:10.1681/ASN.2014111067 (2015).

15 Zilionis, R. *et al.* Single-Cell Transcriptomics of Human and Mouse Lung Cancers Reveals Conserved Myeloid Populations across Individuals and Species. *Immunity* **50**, 1317-1334 e1310, doi:10.1016/j.immuni.2019.03.009 (2019).

16 MacParland, S. A. *et al.* Single cell RNA sequencing of human liver reveals distinct intrahepatic macrophage populations. *Nat Commun* **9**, 4383, doi:10.1038/s41467-018-06318-7 (2018).

17 Mantovani, A., Sozzani, S., Locati, M., Allavena, P. & Sica, A. Macrophage polarization: tumor-associated macrophages as a paradigm for polarized M2 mononuclear phagocytes. *Trends Immunol* **23**, 549-555, doi:10.1016/s1471-4906(02)02302-5 (2002).

18 Sica, A. *et al.* Macrophage polarization in tumour progression. *Semin Cancer Biol* **18**, 349-355, doi:10.1016/j.semcancer.2008.03.004 (2008).

19 Diaz-Montero, C. M., Rini, B. I. & Finke, J. H. The immunology of renal cell carcinoma. *Nat Rev Nephrol* **16**, 721-735, doi:10.1038/s41581-020-0316-3 (2020).

20 Braun, D. A. *et al.* Progressive immune dysfunction with advancing disease stage in renal cell carcinoma. *Cancer Cell* **39**, 632-648 e638, doi:10.1016/j.ccell.2021.02.013 (2021).

21 Vitale, I., Manic, G., Coussens, L. M., Kroemer, G. & Galluzzi, L. Macrophages and Metabolism in the Tumor Microenvironment. *Cell Metab* **30**, 36-50, doi:10.1016/j.cmet.2019.06.001 (2019).
22 Roumenina, L. T. *et al.* Tumor Cells Hijack Macrophage-Produced Complement C1q to Promote Tumor Growth. *Cancer Immunol Res* **7**, 1091-1105, doi:10.1158/2326-6066.CIR-18-0891 (2019).
23 Bi, K. *et al.* Tumor and immune reprogramming during immunotherapy in advanced renal cell carcinoma. *Cancer Cell* **39**, 649-661 e645, doi:10.1016/j.ccell.2021.02.015 (2021).
24 Pearce, D. *et al.* Collecting duct principal cell transport processes and their regulation. *Clin J Am Soc Nephrol* **10**, 135-146, doi:10.2215/CJN.05760513 (2015).
25 Wang, P. *et al.* Dissecting the Global Dynamic Molecular Profiles of Human Fetal Kidney Development by Single-Cell RNA Sequencing. *Cell Rep* **24**, 3554-3567 e3553, doi:10.1016/j.celrep.2018.08.056 (2018).
26 Hou, J., Renigunta, A., Yang, J. & Waldegger, S. Claudin-4 forms paracellular chloride channel in the kidney and requires claudin-8 for tight junction localization. *Proc Natl Acad Sci U S A* **107**, 18010-18015, doi:10.1073/pnas.1009399107 (2010).
27 He, B. *et al.* Single-cell RNA sequencing reveals the mesangial identity and species diversity of glomerular cell transcriptomes. *Nat Commun* **12**, 2141, doi:10.1038/s41467-021-22331-9 (2021).
28 Barry, D. M. *et al.* Molecular determinants of nephron vascular specialization in the kidney. *Nat Commun* **10**, 5705, doi:10.1038/s41467-019-12872-5 (2019).
29 Villani, A. C. *et al.* Single-cell RNA-seq reveals new types of human blood dendritic cells, monocytes, and progenitors. *Science* **356**, doi:10.1126/science.aah4573 (2017).
30 Clark, J. Z. *et al.* Representation and relative abundance of cell-type selective markers in whole-kidney RNA-Seq data. *Kidney Int* **95**, 787-796, doi:10.1016/j.kint.2018.11.028 (2019).
31 Subramanya, A. R. & Ellison, D. H. Distal convoluted tubule. *Clin J Am Soc Nephrol* **9**, 2147-2163, doi:10.2215/CJN.05920613 (2014).
32 Schaefer, L. *et al.* Biglycan, a nitric oxide-regulated gene, affects adhesion, growth, and survival of mesangial cells. *J Biol Chem* **278**, 26227-26237, doi:10.1074/jbc.M210574200 (2003).
33 Chung, J. J. *et al.* Single-Cell Transcriptome Profiling of the Kidney Glomerulus Identifies Key Cell Types and Reactions to Injury. *J Am Soc Nephrol* **31**, 2341-2354, doi:10.1681/ASN.2020020220 (2020).
34 Guessoum, O., de Goes Martini, A., Sequeira-Lopez, M. L. S. & Gomez, R. A. Deciphering the Identity of Renin Cells in Health and Disease. *Trends Mol Med* **27**, 280-292, doi:10.1016/j.molmed.2020.10.003 (2021).
35 Yang, X. *et al.* CD36 in chronic kidney disease: novel insights and therapeutic opportunities. *Nat Rev Nephrol* **13**, 769-781, doi:10.1038/nrneph.2017.126 (2017).
36 Denisenko, E. *et al.* Systematic assessment of tissue dissociation and storage biases in single-cell and single-nucleus RNA-seq workflows. *Genome Biol* **21**, 130, doi:10.1186/s13059-020-02048-6 (2020).
37 Evren, E. *et al.* Distinct developmental pathways from blood monocytes generate human lung macrophage diversity. *Immunity* **54**, 259-275 e257, doi:10.1016/j.immuni.2020.12.003 (2021).
38 Lyons, Y. A., Wu, S. Y., Overwijk, W. W., Baggerly, K. A. & Sood, A. K. Immune cell profiling in cancer: molecular approaches to cell-specific identification. *NPJ Precis Oncol* **1**, 26, doi:10.1038/s41698-017-0031-0 (2017).
39 Borcherding, N. *et al.* Mapping the immune environment in clear cell renal carcinoma by single-cell genomics. *Commun Biol* **4**, 122, doi:10.1038/s42003-020-01625-6 (2021).
40 Aird, W. C. Phenotypic heterogeneity of the endothelium: II. Representative vascular beds. *Circ Res* **100**, 174-190, doi:10.1161/01.RES.0000255690.03436.ae (2007).
41 Krishna, C. *et al.* Single-cell sequencing links multiregional immune landscapes and tissue-resident T cells in ccRCC to tumor topology and therapy efficacy. *Cancer Cell* **39**, 662-677 e666, doi:10.1016/j.ccell.2021.03.007 (2021).

42 Nagao, K. *et al.* Fatty acid binding protein 7 may be a marker and therapeutic targets in clear cell renal cell carcinoma. *BMC Cancer* **18**, 1114, doi:10.1186/s12885-018-5060-8 (2018).

43 Barkal, A. A. *et al.* CD24 signalling through macrophage Siglec-10 is a target for cancer immunotherapy. *Nature* **572**, 392-396, doi:10.1038/s41586-019-1456-0 (2019).

44 Lee, H. J., Kim, D. I., Kwak, C., Ku, J. H. & Moon, K. C. Expression of CD24 in clear cell renal cell carcinoma and its prognostic significance. *Urology* **72**, 603-607, doi:10.1016/j.urology.2008.01.061 (2008).

45 Buffa, F. M., Harris, A. L., West, C. M. & Miller, C. J. Large meta-analysis of multiple cancers reveals a common, compact and highly prognostic hypoxia metagene. *Br J Cancer* **102**, 428-435, doi:10.1038/sj.bjc.6605450 (2010).

46 Zou, Y. *et al.* A GPX4-dependent cancer cell state underlies the clear-cell morphology and confers sensitivity to ferroptosis. *Nat Commun* **10**, 1617, doi:10.1038/s41467-019-09277-9 (2019).

47 Park, J. *et al.* Single-cell transcriptomics of the mouse kidney reveals potential cellular targets of kidney disease. *Science* **360**, 758-763, doi:10.1126/science.aar2131 (2018).

48 Petrovic, S. *et al.* SLC26A7: a basolateral Cl-/HCO3- exchanger specific to intercalated cells of the outer medullary collecting duct. *Am J Physiol Renal Physiol* **286**, F161-169, doi:10.1152/ajprenal.00219.2003 (2004).

49 Kuppe, C. *et al.* Decoding myofibroblast origins in human kidney fibrosis. *Nature* **589**, 281-286, doi:10.1038/s41586-020-2941-1 (2021).

50 Wang, L. *et al.* NDUFA4L2 is associated with clear cell renal cell carcinoma malignancy and is regulated by ELK1. *PeerJ* **5**, e4065, doi:10.7717/peerj.4065 (2017).

51 Verine, J. *et al.* Determination of angptl4 mRNA as a diagnostic marker of primary and metastatic clear cell renal-cell carcinoma. *PLoS One* **5**, e10421, doi:10.1371/journal.pone.0010421 (2010).

52 Jonasch, E., Walker, C. L. & Rathmell, W. K. Clear cell renal cell carcinoma ontogeny and mechanisms of lethality. *Nat Rev Nephrol* **17**, 245-261, doi:10.1038/s41581-020-00359-2 (2021).

53 Szabo, P. A. *et al.* Single-cell transcriptomics of human T cells reveals tissue and activation signatures in health and disease. *Nat Commun* **10**, 4706, doi:10.1038/s41467-019-12464-3 (2019).

54 Azizi, E. *et al.* Single-Cell Map of Diverse Immune Phenotypes in the Breast Tumor Microenvironment. *Cell* **174**, 1293-1308 e1236, doi:10.1016/j.cell.2018.05.060 (2018).

55 Brannon, A. R. *et al.* Molecular Stratification of Clear Cell Renal Cell Carcinoma by Consensus Clustering Reveals Distinct Subtypes and Survival Patterns. *Genes Cancer* **1**, 152-163, doi:10.1177/1947601909359929 (2010).

56 Tran, T. *et al.* In Vivo Developmental Trajectories of Human Podocyte Inform In Vitro Differentiation of Pluripotent Stem Cell-Derived Podocytes. *Dev Cell* **50**, 102-116 e106, doi:10.1016/j.devcel.2019.06.001 (2019).

57 Miller, B. C. *et al.* Subsets of exhausted CD8(+) T cells differentially mediate tumor control and respond to checkpoint blockade. *Nat Immunol* **20**, 326-336, doi:10.1038/s41590-019-0312-6 (2019).

58 Muto, Y. *et al.* Single cell transcriptional and chromatin accessibility profiling redefine cellular heterogeneity in the adult human kidney. *Nat Commun* **12**, 2190, doi:10.1038/s41467-021-22368-w (2021).

59 Hochane, M. *et al.* Single-cell transcriptomics reveals gene expression dynamics of human fetal kidney development. *PLoS Biol* **17**, e3000152, doi:10.1371/journal.pbio.3000152 (2019).

60 Chen, L. *et al.* Renal-Tubule Epithelial Cell Nomenclature for Single-Cell RNA-Sequencing Studies. *J Am Soc Nephrol* **30**, 1358-1364, doi:10.1681/ASN.2019040415 (2019).

61 Rudman-Melnick, V. *et al.* Single-Cell Profiling of AKI in a Murine Model Reveals Novel Transcriptional Signatures, Profibrotic Phenotype, and Epithelial-to-Stromal Crosstalk. *J Am Soc Nephrol* **31**, 2793-2814, doi:10.1681/ASN.2020010052 (2020).

62 Au, L. *et al.* Determinants of anti-PD-1 response and resistance in clear cell renal cell carcinoma. *Cancer Cell* **39**, 1497-1518 e1411, doi:10.1016/j.ccell.2021.10.001 (2021).

63 Yang, C. *et al.* Heterogeneity of human bone marrow and blood natural killer cells defined by single-cell transcriptome. *Nat Commun* **10**, 3931, doi:10.1038/s41467-019-11947-7 (2019).
64 Patil, V. S. *et al.* Precursors of human CD4(+) cytotoxic T lymphocytes identified by single-cell transcriptome analysis. *Sci Immunol* **3**, doi:10.1126/sciimmunol.aan8664 (2018).
