# Supplementary material for: Single-cell transcriptional profiling of clear cell renal cell carcinoma reveals a tumor-associated endothelial tip cell phenotype
Source: Commun Biol. 2024 Jun 28;7:780. doi: 10.1038/s42003-024-06478-x (PMC11213875; doi:10.1038/s42003-024-06478-x)
Supplement: Supplementary file 2 — Description of Additional Supplementary Materials [file 42003_2024_6478_MOESM2_ESM.pdf]

Description of Additional Supplementary Files

File name: Supplementary Data 1

Description: Clinical characteristics of all ccRCC samples used in analysis, related to figure 1c.

File name: Supplementary Data 2

Description: Initial cluster vs rest of cells DGE analysis top 100 fold-changes with adjusted p-value <0.05. Mann Whitney-U with Bonferoni Hochberg correction, related to Supplementary Information table 1

File name: Supplementary Data 3

Description: Refined DGE analysis results used for Figure 1f heatmap, top 15 genes with adjusted p-value <0.05. Mann Whitney-U with Bonferoni Hochberg correction

File name: Supplementary Data 4

Description: Sample composition by broad cell category, related to Figure 1d

File name: Supplementary Data 5

Description: CellPhoneDB results for interaction means between all cell populations in the TME, excluding healthy kidney epithelium and cycling cell populations, related to figures 2c, 3d and 5c

File name: Supplementary Data 6

Description: CellPhoneDB results for interaction p-values between all cell populations in the TME, excluding healthy kidney epithelium and cycling cell populations, related to figures 2c, 3d and 5c

File name: Supplementary Data 7

Description: Cell-cell communication signatures used for survival analysis

File name: Supplementary Data 8

Description: Survival analysis in the TCGA RNA-seq data of gene signature expression using Cox-Wald and Kaplan Meiyer-Log Rank tests.Related to figures 2d, 3e, 4b,c, 5d and Supplementay figures S5b-g.

File name: Supplementary Data 9

Description: DGE results between tumor and healthy endothelium, genes with adjusted p-value <0.05 and log2FC>1 or log2FC<-1 are shown. Mann Whitney-U with Bonferoni Hochberg correction. Related to figure 3b.

File name: Supplementary Data 10

Description: DGE results between endothelial cell populations, top 15 genes with adjusted p-value <0.05. Mann Whitney-U with Bonferoni Hochberg correction. Related to figure 3c.

File name: Supplementary Data 11

Description: Gene set overrepresentation analysis (ORA) in MSigDB Hallmark signatures. Related to figure 4a and Supplementary figure S5a.

File name: Supplementary Data 12

Description: MsigDB EMT pathway overlapping genes for tumor vasculature subpopulations

File name: Supplementary Data 13

Description: DGE results between stromal cell populations, top 20 genes with adjusted p-value <0.05. Mann Whitney-U with Bonferoni Hochberg correction. Related to figure 5b.

File name: Supplementary Data 14

Description: DGE results between tumor and healthy mesangial/vSMC, genes with adjusted p-value <0.05 and log2FC>1 or log2FC<-1 are shown. Mann Whitney-U with Bonferoni Hochberg correction. Related to Supplementary figure S6.
