# Supplementary material for: Single-cell transcriptional profiling of clear cell renal cell carcinoma reveals a tumor-associated endothelial tip cell phenotype
Source: Commun Biol. 2024 Jun 28;7:780. doi: 10.1038/s42003-024-06478-x (PMC11213875; doi:10.1038/s42003-024-06478-x)
Supplement: Supplementary file 4 — Reporting Summary [file 42003_2024_6478_MOESM4_ESM.pdf]

Corresponding author(s): Linas Mazutis
linas.mazutis@bti.vu.lt

Last updated by author(s): May 28, 2024

Reporting Summary

Nature Portfolio wishes to improve the reproducibility of the work that we publish. This form provides structure for consistency and transparency in reporting. For further information on Nature Portfolio policies, see our Editorial Policies and the Editorial Policy Checklist.

Statistics

For all statistical analyses, confirm that the following items are present in the figure legend, table legend, main text, or Methods section.

n/a	Confirmed	
☐	☒	The exact sample size (*n*) for each experimental group/condition, given as a discrete number and unit of measurement
☐	☒	A statement on whether measurements were taken from distinct samples or whether the same sample was measured repeatedly
☐	☒	The statistical test(s) used AND whether they are one- or two-sided<br>*Only common tests should be described solely by name; describe more complex techniques in the Methods section.*
☐	☒	A description of all covariates tested
☐	☒	A description of any assumptions or corrections, such as tests of normality and adjustment for multiple comparisons
☐	☒	A full description of the statistical parameters including central tendency (e.g. means) or other basic estimates (e.g. regression coefficient) AND variation (e.g. standard deviation) or associated estimates of uncertainty (e.g. confidence intervals)
☐	☒	For null hypothesis testing, the test statistic (e.g. *F*, *t*, *r*) with confidence intervals, effect sizes, degrees of freedom and *P* value noted<br>*Give P values as exact values whenever suitable.*
☒	☐	For Bayesian analysis, information on the choice of priors and Markov chain Monte Carlo settings
☒	☐	For hierarchical and complex designs, identification of the appropriate level for tests and full reporting of outcomes
☒	☐	Estimates of effect sizes (e.g. Cohen's *d*, Pearson's *r*), indicating how they were calculated

Our web collection on statistics for biologists contains articles on many of the points above.

Software and code

Policy information about availability of computer code

Data collection	TGCA data was obtained from the NCI GDC Data Portal using the TCGAbiolinks R package (v2.24.3). The exact code of how the data was obtained is provided at https://github.com/zvirblyte/2023_ccRCC/blob/main/R/prepare_data.R
Data analysis	Data analysis was performed using Python and R, all notebooks are provided at https://github.com/zvirblyte/2023_ccRCC.

For manuscripts utilizing custom algorithms or software that are central to the research but not yet described in published literature, software must be made available to editors and reviewers. We strongly encourage code deposition in a community repository (e.g. GitHub). See the Nature Portfolio guidelines for submitting code & software for further information.

Data

Policy information about availability of data

All manuscripts must include a data availability statement. This statement should provide the following information, where applicable:

- Accession codes, unique identifiers, or web links for publicly available datasets
- A description of any restrictions on data availability
- For clinical datasets or third party data, please ensure that the statement adheres to our policy

The data generated in this study are available in Gene Expression Omnibus (GEO) at GSE242299. Publicly available datasets used were downloaded from GEO (at GSE159115) and https://endotheliomics.shinyapps.io/lung_ectax/. TCGA KIRC cohort bulk RNA-seq (upper quartile FPKM normalized) and clinical data were downloaded from the NCI GDC Data Portal using the TCGAbiolinks R package.

Research involving human participants, their data, or biological material

Policy information about studies with human participants or human data. See also policy information about sex, gender (identity/presentation), and sexual orientation and race, ethnicity and racism.

Reporting on sex and gender	Sex of the participants is reported, but no scRNA-seq data analysis with regards to sex was performed.
Reporting on race, ethnicity, or other socially relevant groupings	N/A
Population characteristics	Characteristics including age, sex, histological tumor subtype, pT stage, tumor size, ISUP/WHO differentiation grade, extrarenal extension, tumor invasion to renal pelvis, tumor invasion to renal vein, lymphovascular invasion, fat invasion and necrosis were collected for every patient enrolled in the study.
Recruitment	Patient with confirmed clear cell renal carcinoma disease with no prior systemic therapy were recruited.
Ethics oversight	Vilnius regional bioethics committee approval No.2019/2-1074-586.

Note that full information on the approval of the study protocol must also be provided in the manuscript.

Field-specific reporting

Please select the one below that is the best fit for your research. If you are not sure, read the appropriate sections before making your selection.

☒ Life sciences ☐ Behavioural & social sciences ☐ Ecological, evolutionary & environmental sciences

For a reference copy of the document with all sections, see nature.com/documents/nr-reporting-summary-flat.pdf

Life sciences study design

All studies must disclose on these points even when the disclosure is negative.

Sample size	9 patients.
Data exclusions	Tumor sample from patient P1 was excluded from analysis due to high level of necrosis in tissue.
Replication	N/A
Randomization	Patients fulfilling inclusion criteria (confirmed clear cell renal carcinoma disease with no prior systemic therapy) we randomly enrolled to the study.
Blinding	N/A

Reporting for specific materials, systems and methods

We require information from authors about some types of materials, experimental systems and methods used in many studies. Here, indicate whether each material, system or method listed is relevant to your study. If you are not sure if a list item applies to your research, read the appropriate section before selecting a response.

Materials & experimental systems

n/a	Involved in the study	
☒	☐	Antibodies
☒	☐	Eukaryotic cell lines
☒	☐	Palaeontology and archaeology
☒	☐	Animals and other organisms
☒	☐	Clinical data
☒	☐	Dual use research of concern
☒	☐	Plants

Methods

n/a	Involved in the study	
☒	☐	ChIP-seq
☒	☐	Flow cytometry
☒	☐	MRI-based neuroimaging

Plants

Seed stocks	n/a
Novel plant genotypes	n/a
Authentication	n/a
